# Chitosan overlaid Fe_3_O_4_/rGO nanocomposite for targeted drug delivery, imaging, and biomedical applications

**DOI:** 10.1038/s41598-020-76015-3

**Published:** 2020-11-03

**Authors:** Viswanathan Karthika, Mohamad S. AlSalhi, Sandhanasamy Devanesan, Kasi Gopinath, Ayyakannu Arumugam, Marimuthu Govindarajan

**Affiliations:** 1 Department of Packaging, Yonsei University, 1 Yonseidae-gil, Wonju-si, Gangwon-do 26493 South Korea; 2 Research Chair in Laser Diagnosis of Cancers, College of Science, Department of Physics and Astronomy, King Saud University, Riyadh, 11451 Kingdom of Saudi Arabia; 3 Department of Physics and Astronomy, College of Science, King Saud University, Riyadh, 11451 Kingdom of Saudi Arabia; 4 Department of Botany, Alagappa University, Karaikudi, Tamil Nadu 630 003 India; 5 Department of Zoology, Unit of Vector Control, Phytochemistry and Nanotechnology, Annamalai University, Annamalainagar, Tamil Nadu 608 002 India; 6 Department of Zoology, Unit of Natural Products and Nanotechnology, Government College for Women (Autonomous), Kumbakonam, Tamil Nadu 612 001 India

**Keywords:** Biophysics, Cancer, Chemical biology, Drug discovery, Materials science, Nanoscience and technology

## Abstract

A hybrid and straightforward nanosystem that can be used simultaneously for cancer-targeted fluorescence imaging and targeted drug delivery in vitro was reported in this study. A chitosan (CS) polymer coated with reduced graphene oxide (rGO) and implanted with Fe_3_O_4_ nanoparticles was fabricated. The fundamental physicochemical properties were confirmed via FT-IR, XRD, FE-SEM, HR-TEM, XPS, and VSM analysis. The in vivo toxicity study in zebrafish showed that the nanocomposite was not toxic. The in vitro drug loading amount was 0.448 mg/mL^−1^ for doxorubicin, an anticancer therapeutic, in the rGO/Fe_3_O_4_/CS nanocomposite. Furthermore, the pH-regulated release was observed using folic acid. Cellular uptake and multimodal imaging revealed the benefit of the folic acid-conjugated nanocomposite as a drug carrier, which remarkably improves the doxorubicin accumulation inside the cancer cells over-express folate receptors. The rGO/Fe_3_O_4_/CS nanocomposite showed enhanced antibiofilm and antioxidant properties compared to other materials. This study's outcomes support the use of the nanocomposite in targeted chemotherapy and the potential applications in the polymer, cosmetic, biomedical, and food industries.

## Introduction

One current focus of the scientific community is combating cancer using nanotherapeutics, chemotherapy, and gene therapy. Current strategies for the drug delivery of cancer therapeutics are focused on increasing the drug concentration at the target site and reducing the systemic distribution. In this regard, functionalized nanoparticles (NPs) are of interest because they can prevent the systemic metabolism and subsequent elimination of the drug, thus ensuring pharmacological effect with less toxicity^1^. Advancements in nanoscience have facilitated the selective transportation of drugs into the target site by a unique mechanism (ligand-mediated targeting and receptor-mediated targeting molecules)—this makes to deliver the specific antitumor molecule to the cancer tissue. The sensitive nanocarriers are size- and dimension-dependent. After exposure to an external stimulus, such as pH, enzymatic systems, magnetic gradient, temperature, and ultrasound, the nanocarriers change their physicochemical properties^1,2^. Superparamagnetic Fe_3_O_4_ NPs have gained attention for their multifunctionality, including target-based carriage, localized hyperthermia therapy, stem cell labeling and tracking, and contrast agents for magnetic resonance imaging (MRI)^3–6^.

Additionally, nanocomposites with these superparamagnetic NPs and a 2-dimensional graphene derivative with a large surface area can be used to rational design the drug delivery system. Thus, nanocomposites with a high drug loading capacity and magnetically controlled carrier can be accomplished simultaneously. The preferred graphene derivative for drug loading and delivery efficiency is graphene oxide (GO), because its viable surface chemistry contains layers of graphene sheets with various organic functional groups, such as carboxylic acid, epoxide, and hydroxide, on its surface^7^. The peripheral carboxylic group (–COO) can stabilize the colloidal system and create a pH-responsive negative charge surface. However, the basal plane having hydroxyl (–OH) and the epoxide (–O^−^) groups results in the weak chemical interaction, such as H-bonding, or chemical interaction causing the transpire on the surface^8–11^. Even the entire areas of GO can be used for drug loading and non-covalent functionalization because of the possible π–π interactions on the basal plane. Thus, when designing an efficient drug delivery system, GO acts as a surfactant to stabilize hydrophobic molecules due to its amphiphilic nature^12,13^. However, Wang et al.^14^ reported that pure GO is toxic to cells in vitro and animals, as the kidneys cannot empty it, thereby resulting in the granuloma formation in the lung or cell apoptosis lung granuloma formation even though apparent toxicity did not observe in the lower dose and moderate dose approximately 0.1 mg and 0.25 mg, respectively. Thus, GO must be coated with polymers, such as chitosan (CS), and polyethylene glycol, to improve its solubility. CS is made of two types of structural units: 2-amino-2-deoxy-d-glucose and N-acetyl-2-amino-2-deoxy-d-glucose linked by a β(1 → 4) bond, and CS is derived from chitin^15^. CS is a polycationic biopolymer, abundant in different crustacean shells like crab, shrimp and crawfish. CS is a low-cost material with biodegradable and biocompatible properties, and ease of chemical modification. CS posses chelation property and selectively binds with metal ions and biomolecules like proteins, cholesterol and tumor cells. Due to this property, CS is widely used in the food industry, pharmaceutical, and water management farms. CS possesses properties useful in the medical field to inhibit tumor cells, as antimicrobial agents, as a wound-healing agent and as immunostimulant^16^. CS-coated GO has improved solubility and drug loading capability, in addition to its increased potential to distribute the drug molecule at the tumor site, which has an acidic environment^7,9,15^.

When evaluating the designed drug delivery system's efficacy, the loaded drugs and ligands should be considered. Doxorubicin (DOX) is one of the best therapeutic agents for cancers. The cytotoxicity and DNA cleavage can be occurred through binding to DNA (intercalation mode) or reacting with topoisomerase II^16,17^. Folic acid (FA) is a highly selective ligand that binds to the folate receptor^18^. This ligand also has high target specificity to various types of cancer cells. Binding of the carrier molecules with FA improves the efficiency and reduces the adverse effects of the drug molecules^19,20^. The pH-responsive release behavior and anticancer activity of Dox-loaded, FA-conjugated, GO-Fe_3_O_4_ nanocomposites were reported in the previous studies. However, The combination of rGO/Fe_3_O_4_/CS was a novel attempt. Such a facile protocol was resulted in Fe_3_O_4_ with a size of less than 35 nm. This combination can be applied to the drug delivery system because it presented an appropriate cancer therapy result. Briefly, rGO can interact with DOX through a π–π bond and hence enhance the drug loading capacity, in which Fe_3_O_4_, as a magnetic agent, can readily move through the therapy without any external magnetic field. Besides, the biopolymeric backbone of nanocomposite (CS) can control the neutral pH's release behavior.

Furthermore, FA is a receptor and DOX is a model drug. Therefore, the current attempt was carried out as a novel study, in which there has been no report on trGO/Fe_3_O_4_/CS/FA/DOX combination. Accordingly, this study aimed to achieve efficient drug loading, release, and fluorescence imaging with chemo-photothermal combination therapy for cancer; herein, we report a biocompatible theragnostic nano platform, rGO/Fe_3_O_4_/CS, which was synthesized via a solvothermal method and studied as a targeted drug delivery system with DOX and FA (DOX/rGO/Fe_3_O_4_/CS/FA).

## Results and discussion

### Chemical bond analysis using FT-IR

**Figure 1:**
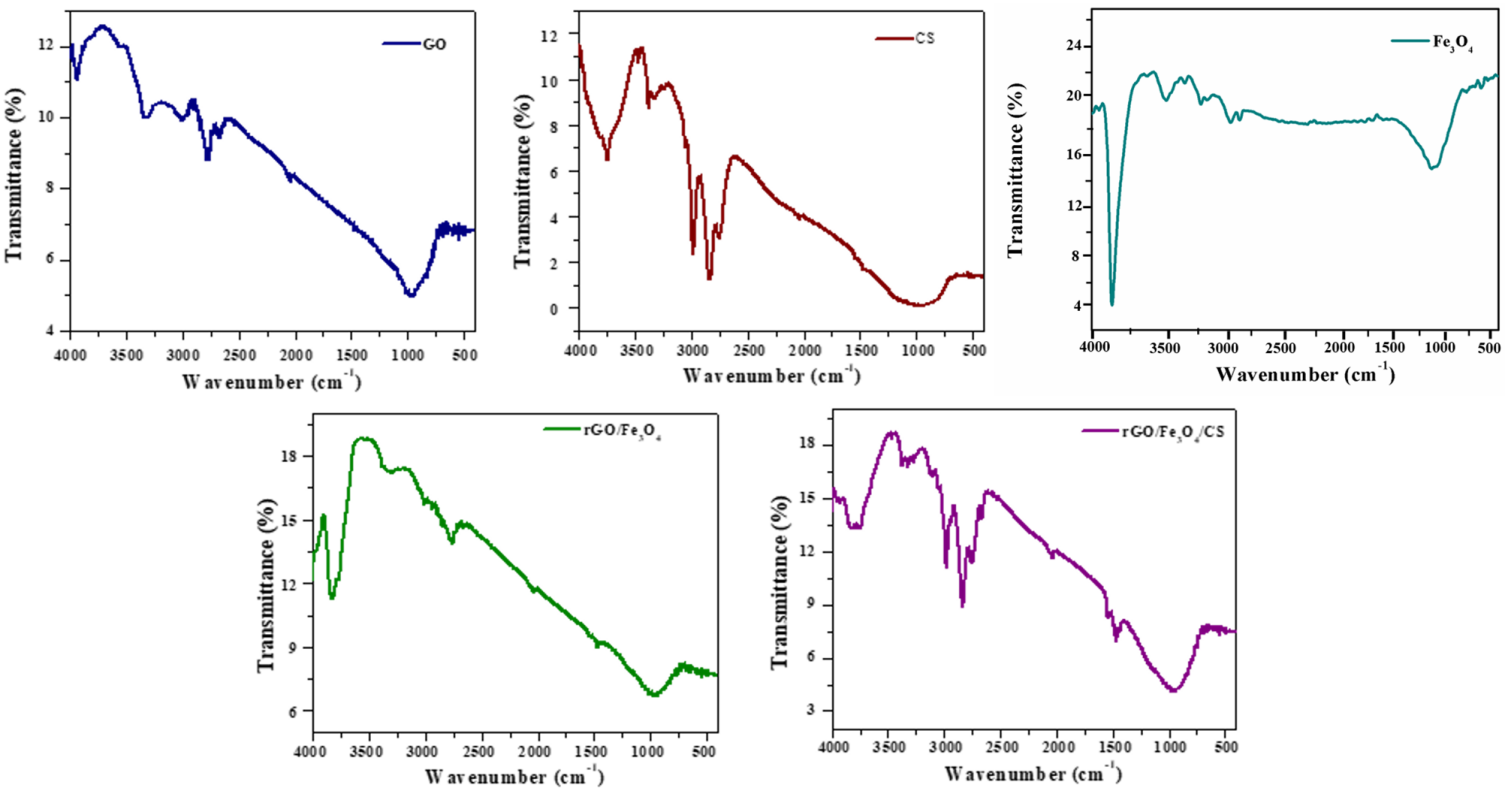
Fourier transformed-infrared (FT-IR) spectra of graphene oxide (GO), chitosan (CS), Fe_3_O_4_, rGO/Fe_3_O_4_ (5%), and rGO/Fe_3_O_4_/CS (15%) nanocomposites.

The chemical properties and bonding nature of the synthesized rGO/Fe_3_O_4_ (5%) and rGO/Fe_3_O_4_/CS (15%) nanocomposites were studied by infrared spectroscopy, and the obtained spectra are shown in Fig. 1. The nanocomposites' spectra were primarily compared with the FT-IR spectra of pure GO, CS, and Fe_3_O_4_ NPs (Fig. 1) to ensure the presence of the respective materials in both nanocomposites. The peaks present at 3563 cm^−1^, 2036 cm^−1^, 1410 cm^−1^, and 959 cm^−1^ in the spectra of the pure GO corresponds to the O–H, C=O, C–OH, and C–O functional groups, respectively, (Fig. 1). In pure CS, peaks at 968 cm^−1^, 2006 cm^−1^, and 2975 cm^−1^ corresponded to the characteristic stretching vibrations of C–O, NH_2_, and C–H bonds, respectively (Fig. 1)^21^. A common broad peak at 3378 cm^−1^ present in the FT-IR spectra of both GO and CS was credited to the hydroxyl groups present in the adsorbed water molecules present at the surface of both GO and CS. In the Fe_3_O_4_ NP, the peak at 604 cm^−1^ represented the presence of a Fe–O bond in the material (Fig. 1)^20,22^. Furthermore, the peak at 604 cm^−1^ attributed to the stretching vibration of the Fe–O bond. This band was shifted to 952 cm^−1^, due to the bonding of Fe_3_O_4_ NPs with the –COO– groups on the rGO surface^23^. The FT-IR spectra of the nanocomposites showed a synergistic effect between the pure GO and the Fe_3_O_4_ NPs on the properties of CS. In the IR spectrum of GO, Fe_3_O_4_ NPs, CS, the peaks at 3933, 3322, 2975, 2840, 2662, 2036, 1634, 1440, 968 and 604 cm^−1^ shifted to 3940, 3329, 2984, 2835, 2655, 2045, 1545, 1473, and 952 cm^−1^, respectively, which might exhibit the successful formation of rGO/Fe_3_O_4_/CS nanocomposites. The positive shift of the –OH peak in the rGO/Fe_3_O_4_/CS composite indicated an H-bond interaction between fillers and the CS (Fe_3_O_4_ and GO)^24^.

### Crystal structural analysis using XRD

**Figure 2:**
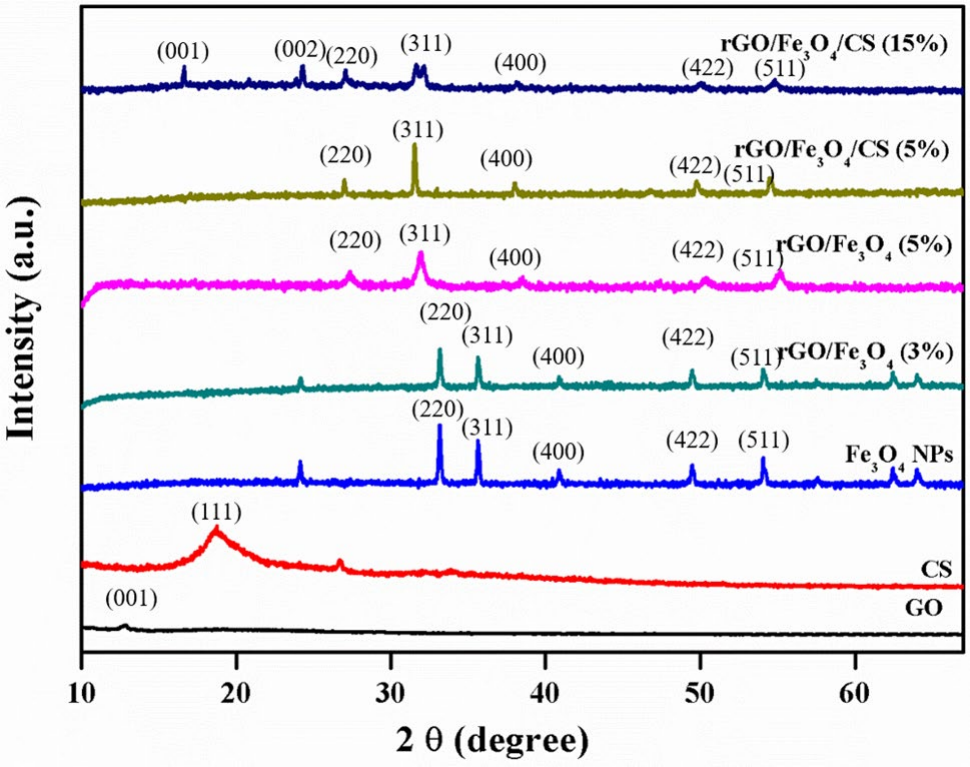
X-ray diffraction (XRD) pattern of graphene oxide (GO), chitosan (CS), Fe_3_O_4_, rGO/Fe_3_O_4_ (3%), rGO/Fe_3_O_4_ (5%), rGO/Fe_3_O_4_/CS (5%), and rGO/Fe_3_O_4_/CS (15%) nanocomposites.

As shown in Fig. 2, the crystallographic properties of both rGO/Fe_3_O_4_ and rGO/Fe_3_O_4_/CS nanocomposites were obtained using X-ray powder diffraction (XRD) patterns. A sharp peak at 2θ = 12.7° and a broad peak at 2θ = 25.9° corresponding to the (001) and (002) planes, respectively, confirmed the formation of GO with an interlayer thickness of about 8.75 Å and weak amorphous peak at 2θ = 19.2° could be attributed to CS as shown in Fig. 2^25^. The peaks present at the 2θ values, 30.07°, 35.4°, 37.05°, 43.05°, 53.41°, 56.93°, and 62.52°. Both the XRD patterns appeared due to the diffraction of X-ray by the (220), (311), (222), (400), (422), (333), and (440) planes, respectively. It is apparent evidence for the existence of the Fe_3_O_4_ phase in both nanocomposites. The Scherrer equation was used to calculate the average crystalline size of the Fe_3_O_4_ NPs in the rGO/Fe_3_O_4_ (20, 37) and rGO/Fe_3_O_4_/CS (60 nm)^26^. The obtained Fe_3_O_4_ phase correlated well with the standard JCPDS card No: 82–1533 with a face-centered cubic crystal structure. The diffraction pattern of both composites' intensity was low and peaks shifted corresponding to GO/rGO or CS. It may be due to the crystalline nature of rGO or CS, which has sharp diffraction peaks that suppress the low intensity of Fe_3_O_4_ NPs peaks.

### FE-SEM analysis

**Figure 3:**
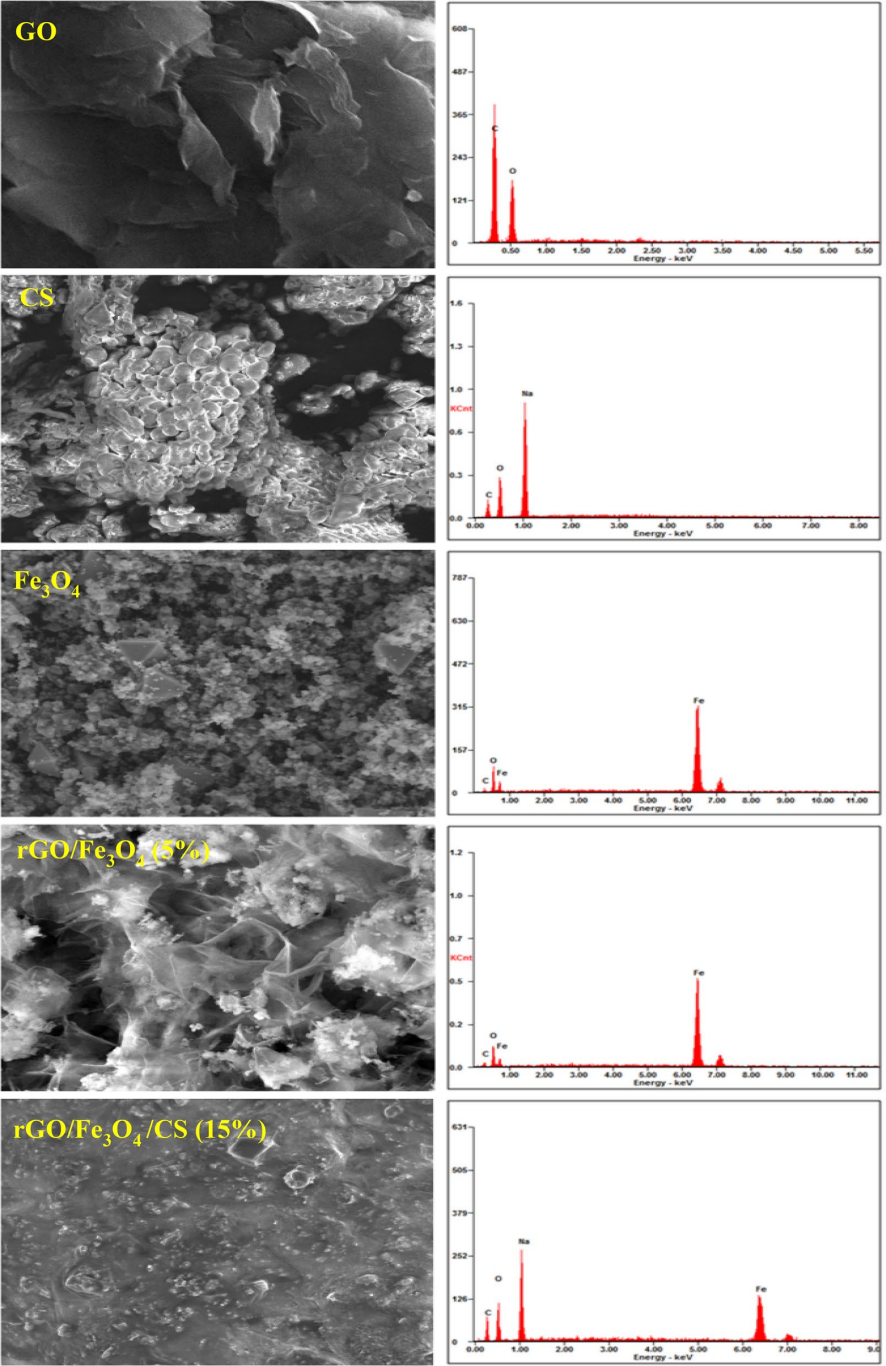
Field emission-scanning electron microscopy (FE-SEM) images and corresponding EDS spectra of graphene oxide (GO), chitosan (CS), Fe_3_O_4_, rGO/Fe_3_O_4_ (5%), and rGO/Fe_3_O_4_/CS (15%) nanocomposites.

The GO morphology was observed using field emission -scanning electron microscopy (FE-SEM), by which GO illustrated a network structure with a high specific surface area and minimum thickness formed by the GO nanosheets (Fig. 3). In pure CS (Fig. 3), smaller particles were agglomerated and formed large clusters with irregular shapes. The pure Fe_3_O_4_ NPs had a spherical shape with a particle size distribution of 20–25 nm, and seemed to be well distinguishable and mostly agglomerate-free. These properties are essential to attain the superparamagnetic property. The morphological changes in both GO nanosheets and Fe_3_O_4_ NPs during the formation of the rGO/Fe_3_O_4_ (5%) nanocomposite were visible in their FE-SEM images, as shown in Fig. 3. The reduction of GO to rGO produced more thin and transparent sheets due to the additional exfoliation of the sheets during the removal of oxygen species.

The Fe_3_O_4_ NPs with a size distribution of 20–25 nm were encapsulated in the interconnected networks of the rGO sheets. The open edges of the rGO attached to the Fe_3_O_4_ NPs via functional groups during the formation of rGO/Fe_3_O_4_ nanocomposite. These interactions tend to strongly prevent the sheets' agglomeration in the matrix and improve the active surface for efficient drug loading and release. The rGO/Fe_3_O_4_/CS (15%) nanocomposite showed aggregated particles on the surface of GO, increasing the size. This feature is due to the agglomeration of Fe_3_O_4_ NPs and the changes introduced by CS. After the addition of CS, the particles attached more tightly to the surface of the rGO sheet. Furthermore, the energy-dispersive x-ray spectroscopy (EDX) spectra of all of the samples confirmed the presence of the essential elements in the respective materials, as shown in Fig. 3.

### HR-TEM analysis

**Figure 4:**
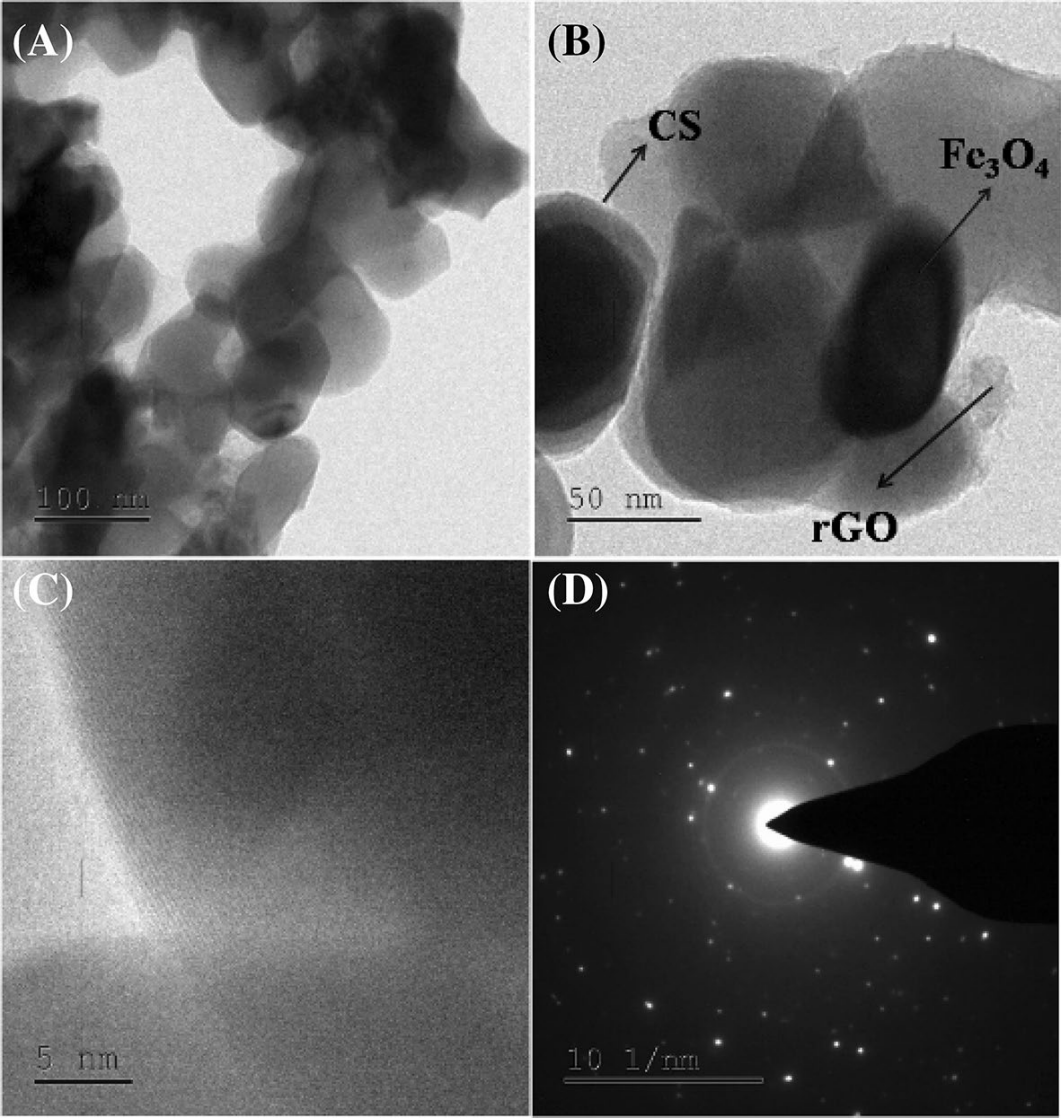
(A-C) High resolution-transmission electron microscopy (HR-TEM) images of rGO/Fe_3_O_4_/CS (15%) nanocomposites and (D) selected area electron diffraction (SAED) pattern of rGO/Fe_3_O_4_/CS (15%) nanocomposites.

As shown in Fig. 4A,B, the rGO/Fe_3_O_4_/CS (15%) nanocomposite was evaluated using the high-resolution transmission electron microscopy (HR-TEM), in which images indicated a homogeneous distribution of Fe_3_O_4_ and CS with a distorted spherical morphology on the randomly stacked rGO sheet surface. The size distribution of the Fe_3_O_4_ NPs and CS attached to the rGO sheets was 25–35 nm. The individual Fe_3_O_4_ and CS particles on the rGO surface were distinguishable, as shown in Fig. 4. The intermediate region of Fe_3_O_4_, CS, and rGO was uniformly and tightly attached to the rGO sheet's surface, as shown in the HR-TEM images presented in Fig. 4C. The rGO/Fe_3_O_4_/CS (15%) composites' lattice spacing was measured as 0.25 nm, which corresponds to the (311) plane of the Fe_3_O_4_ crystal system face-centered cubic system, as shown in the HR-TEM images^27^. The selected area electron diffraction further investigated the crystalline morphology of Fe_3_O_4_ NPs incorporated in the nanocomposite (15%). (SAED) (Fig. 4D). The crystalline plane of the Fe_3_O_4_ system was detected by its diffraction dots and rings represented, which is in agreement with the XRD data.

### Chemical state analysis using XPS

**Figure 5:**
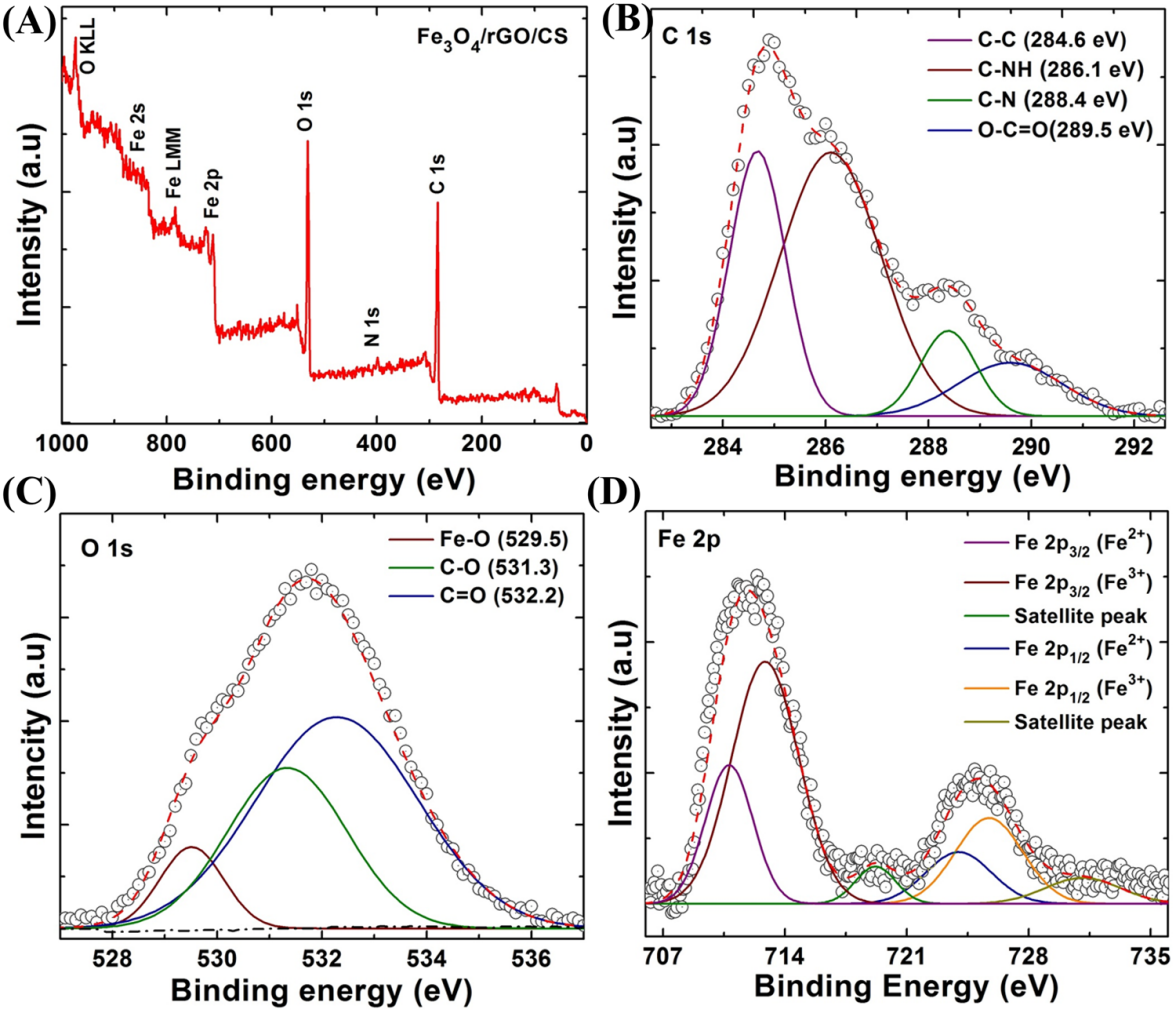
X-ray photoelectron spectroscopy (XPS) survey spectra of rGO/Fe_3_O_4_/CS (15%) nanocomposites (**A**)—high-resolution spectra of rGO/Fe_3_O_4_/CS, (**B**)—C 1 s peak, (**C**)—O 1 s peak, and (**D**)—Fe 2p peak.

The chemical composition, oxidation states of individual elements, and nature of the intermediate bonding between materials in the Fe_3_O_4_/rGO/CS (15%) nanocomposite were studied using X-ray photoelectron spectroscopy (XPS), and the spectra are shown in Fig. 5A–D. The full-scale survey spectrum indicated the presence of iron, carbon, oxygen, and nitrogen from their characteristic Fe 2p, C 1 s, O 1 s, and N 1 s peaks with binding energy values of 725.3, 284.5, 531.5, and 399.8 eV respectively^20^ (Fig. 5A). The presence of the Fe 2p peak in the survey spectra was from the Fe_3_O_4_ NPs, whereas the N 1 s peak might have been from the CS group in the composite. The O 1 s peak was due to the oxygen species present in all of the three materials. Figure 5D illustrates the high-resolution XPS spectra of Fe 2p. The Fe 2p peak was further deconvoluted into 6 individual peaks, in which the peaks present at 710.8 and 723.9 eV emerged due to the 2p_3/2_ and 2p_1/2_ split orbitals of the Fe^2+^ ions, respectively. The peaks at 712.8 and 725.7 eV were due to the 2p_3/2_ and 2p_1/2_ split orbitals of Fe^3+^ ions. The Fe_3_O_4_ phase in the prepared nanocomposite was confirmed by the presence of the Fe^3+^ and Fe^2+^ ions.

In the Fe_3_O_4_ phase, two different Fe sites, which can be represented as [Fe^3+^]A and [Fe^3+^, Fe^2+^]BO_4_, were present. The complete tetrahedral A site and partial octahedral B sites were occupied by Fe^3+^ ions, whereas the Fe^2+^ ions occupied only half of the octahedral B. The two small additional peaks present at 719.2 and 731.2 eV represented the satellite peaks of Fe 2p_3/2_ and Fe 2p_1/2_. Among the four deconvoluted peaks of the C 1 s spectra shown in Fig. 5B, the peak at 284.6 eV appeared due to the C–C and C=C species in rGO sheets and cytosine, whereas the peak at 286.1 and 288.4 eV corresponded to the C–NH and C–N bonds present in CS, respectively. A broad peak present at 289.5 eV was a result of the O–C=O bond. The high-resolution O 1 s spectra were further deconvoluted to three individual peaks (Fig. 5C). The peak appearing at 529.5 eV was a result of the oxygen atoms bound to Fe in Fe_3_O_4_. The other two peaks at 531.3 and 532.2 eV represented C–O and C=O, respectively, in rGO and CS.

### Magnetic measurements using VSM

**Figure 6:**
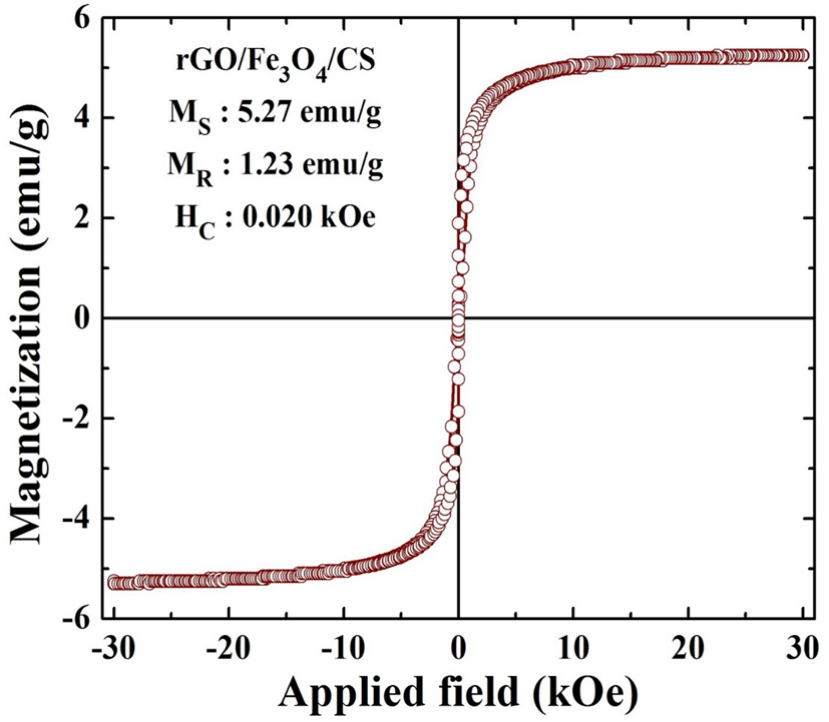
Vibrating sample magnetometer (VSM) analysis of rGO/Fe_3_O_4_/CS (15%) nanocomposites.

Figure 6 illustrates the M-H loop of the rGO/Fe_3_O_4_/CS (15%) nanocomposite under a maximum induced magnetic field of ± 30 kOe at room temperature. The prepared nanocomposite was superparamagnetic with a saturation magnetization (MS) of 5.27 emu/g, attributed to Fe_3_O_4_ NPs in the composition^8,20^. However, it should be noted that the obtained MS value was low compared to that of pure Fe_3_O_4_ NPs according to the previous reports^28^. This significant decrease in the MS of rGO/Fe_3_O_4_/CS (15%) nanocomposite may have been due to the existence of a high amount of non-magnetic rGO and CS^29^. However, the prepared composite showed excellent response in the external magnetic field. The other magnetic parameters, such as remanent magnetization (MR) and coercive field (HC), were 1.23 emu/g and 0.020 kOe. Hence, it can be concluded that the presence of Fe_3_O_4_ NPs possesses a remarkable effect on the superparamagnetic characteristics of the prepared rGO/Fe_3_O_4_/CS (15%) nanocomposite.

### Pharmacological studies

#### In vivo toxicity study of GO, CS, Fe_3_O_4_, rGO/Fe_3_O_4_, and rGO/Fe_3_O_4_/CS nanocomposite in zebrafish embryos

**Figure 7:**
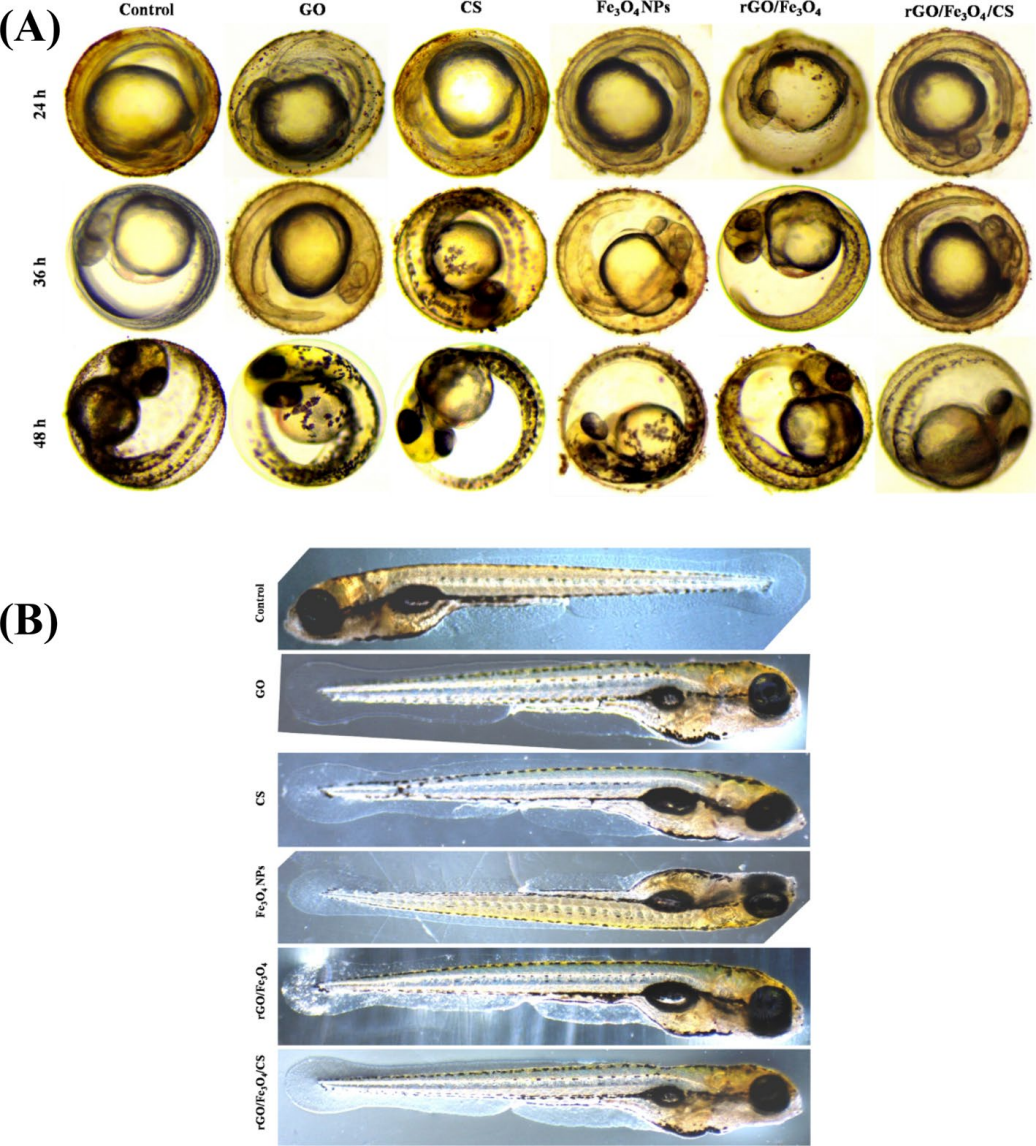
(**A**)—Microscopy images taken after microinjection in zebrafish embryos exposed to 30 µg/mL of graphene oxide (GO), chitosan (CS), Fe_3_O_4_, rGO/Fe_3_O_4_ (5%), and rGO/Fe_3_O_4_/CS (15%) for (24, 36, 48 h). (**B**)—Microscopy images of zebrafish chorions with and without nanoparticles (NPs) treatment (0.1 ng/nL, 72 h).

To assess the biocompatibility and in vivo toxicity of the GO, CS, Fe_3_O_4_, rGO/Fe_3_O_4_, and rGO/Fe_3_O_4_/CS nanocomposite, zebrafish *Danio rerio* (*D. rerio*] was chosen as a model animal, due to early embryonic improvement during 120 h, apparent transparency, a similarity with mammals, and easy maintenance^30,31^. The zebrafish embryos were treated with approximately of 0.1 ng/nL nanomaterials at the 2-cell stage. The embryos were maintained until complete development (72 hpf, hours post fertilization) (Fig. 7).

Additionally, the nanocomposite's different material combinations did not impact the survival rate of zebrafish at various developmental stages (from 0 to 72 h), as shown in Fig. 7. This finding was related to the utilization of CS by the embryo as nourishment. Interestingly, our results did not exhibit any abnormalities compared with other materials. Earlier, toxicological studies on mice have presented that graphene strongly improves the dispersity in the physiological medium and enhances biocompatibility^32,33^. In the present study, rGO/Fe_3_O_4_/CS exhibited less toxicity and malformations in zebrafish.

#### In vitro studies on drug loading and pH-regulated release properties of rGO/Fe_3_O_4_/CS/FA

**Figure 8:**
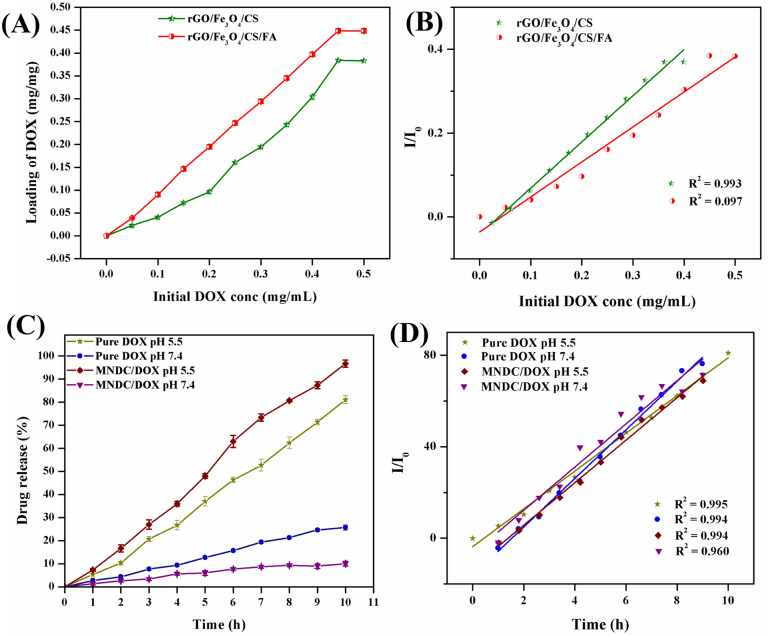
(**A**)—Cumulative amount of DOX loading on rGO/Fe_3_O_4_/CS and rGO/Fe_3_O_4_/CS/FA nanocomposites, (**B**)–Linear fitting of the cumulative amount of DOX loading concentration, (**C**)—Cumulative percentage of pure DOX and rGO/Fe_3_O_4_/CS/FA/DOX drug releasing at different pH, (**D**)—Linear fitting of the cumulative percentage of drug release at different pH (P < 0.05).

The loading amount of DOX in the rGO/Fe_3_O_4_/CS/FA nanocomposite was estimated from its characteristic UV–Vis spectrum at 233 nm by comparing the original DOX concentrations rGO/Fe_3_O_4_/CS/FA nanocomposite solution to that of the supernatant after loading. Figure 8A shows the images of the rGO/Fe_3_O_4_/CS/FA nanocomposite loaded with DOX in necessary conditions (pH 7.4). A maximum of 95% of DOX molecules was loaded on the rGO/Fe3O4/CS/FA nanocomposite under necessary conditions based on the studies. The saturated loading amount of DOX in the rGO/Fe_3_O_4_/CS/FA nanocomposite was 0.5 mg/mL^−1^; therefore, a maximum of 0.448 mg/mL^−1^ of DOX was loaded^34^. Sasikala et al.^34^ claimed the highest loaded amount of drugs on the graphene. However, in this study, the loaded drug amount was higher than that of the reported value. This increased concentration of loaded DOX may be attributed to three significant phenomena: (i) π–π stacking and the H-bonding between GO and DOX (ii) the interaction between the phenolic OH and alkaline amino groups of the DOX molecule with the amino groups and hydroxy on the CS shell of the rGO/Fe_3_O_4_/CS/FA nanocomposite, which forms intermolecular complexes by hydrogen bonding; (iii) and the mucus nature of the CS shell, which provided physical adsorption, the rGO, which provided high surface volume binding with the OH group, and the superparamagnetic materials, which results in high drug loading efficiency of the synthesized nanocomposite. Although Fe_3_O_4_ NPs occupied some surface area of GO, a comparable amount of DOX was loaded. This amount of loaded DOX was higher than that found in commonly used nanocarriers, such as polymer micelles, hydrogel microparticles, liposomes, and carbon nanohorns, where the loading amount is reported as below 1 mg/mg^−1^^35–38^. Additionally, the DOX molecules loaded under necessary conditions were highly efficient and reliable for target delivery because the free carboxylic acid groups can form hydrogen bonds, thus resulting in efficient pH-induced targeted delivery. Additionally, the prepared rGO/Fe_3_O_4_/CS/FA nanocomposite showed a homogenous dispersion in an aqueous medium due to the carboxylic and hydroxyl groups' existence graphene.

Furthermore, the drug release at 37 °C from the composite was studied in simulated body fluid (phosphate-buffered saline; PBS, pH 7.4) and pH 5.5 (PBS). The release profile of DOX from the rGO/Fe_3_O_4_/CS/DOX/FA nanocomposite into the outer aqueous phase is shown in Fig. 8B. The faster pure DOX release behavior at acidic condition was detected, which was pH-dependent, in which the release value was 25% at pH 7.4 and 81% at pH 5.5. Such a pH-responsive property of MNDC can be practically utilized as a controlled release system, which can gently release doxorubicin in the cancer cell because the pH of intracellular is lower than healthy cells^39^. Under the two different pH conditions, the cumulative drug release prominently occurred at 10 h, and maximum (96.6% and 10%) drug release was achieved at pH 5.5 and at pH 7.4, respectively. This decrease in drug release at pH 7.4 may be due to DOX's decreased solubility at pH 7.4.

Additionally, the CS polymer was less soluble at pH 7.4, resulting in low permeability. The biodegradable combination of rGO with CS has presented stronger mechanical properties with a large drug delivery capacity, considered a pH-responsive and biodegradable drug delivery system. CS NPs conjugated with drug via pH-cleavable interaction tend to dissociate at the acid condition of endo-lysosomes, and then release the drug into the cytoplasm. The increment in the biodistribution level and drug concentration in the cancer cells is the CS's major anticancer mechanism. CS incorporated with mifepristone controlled the drug release in a constant release behavior and improved the drug's oral bioavailability and anticancer properties via pharmacokinetic study under in vivo analysis^40^. At pH 5.5, the release of DOX was favorable, as the CS polymer was water-soluble, resulting in increased water content and increased permeability of DOX. Because of this pH dependence, DOX can be specifically distributed around tumor tissues rather than non-selective release throughout the body. The pH-dependent release of anticancer drugs was high in acidic conditions. Thus, DOX molecules were protonated and the hydrophobic interactions between DOX molecules and the nanocomposites were decreased in an acidic environment, which accelerated the DOX release. The protonation of DOX molecules and reduced hydrophobic interactions between DOX molecules and the nanocomposites were observed at acidic conditions, which accelerated the DOX release. It demonstrates the potential use of the rGO/Fe_3_O_4_/CS/DOX/FA nanocomposite in cancer therapy^41^.

#### In vitro cytotoxicity studies of the rGO/Fe_3_O_4_/CS/DOX/FA nanocomposite

**Figure 9:**
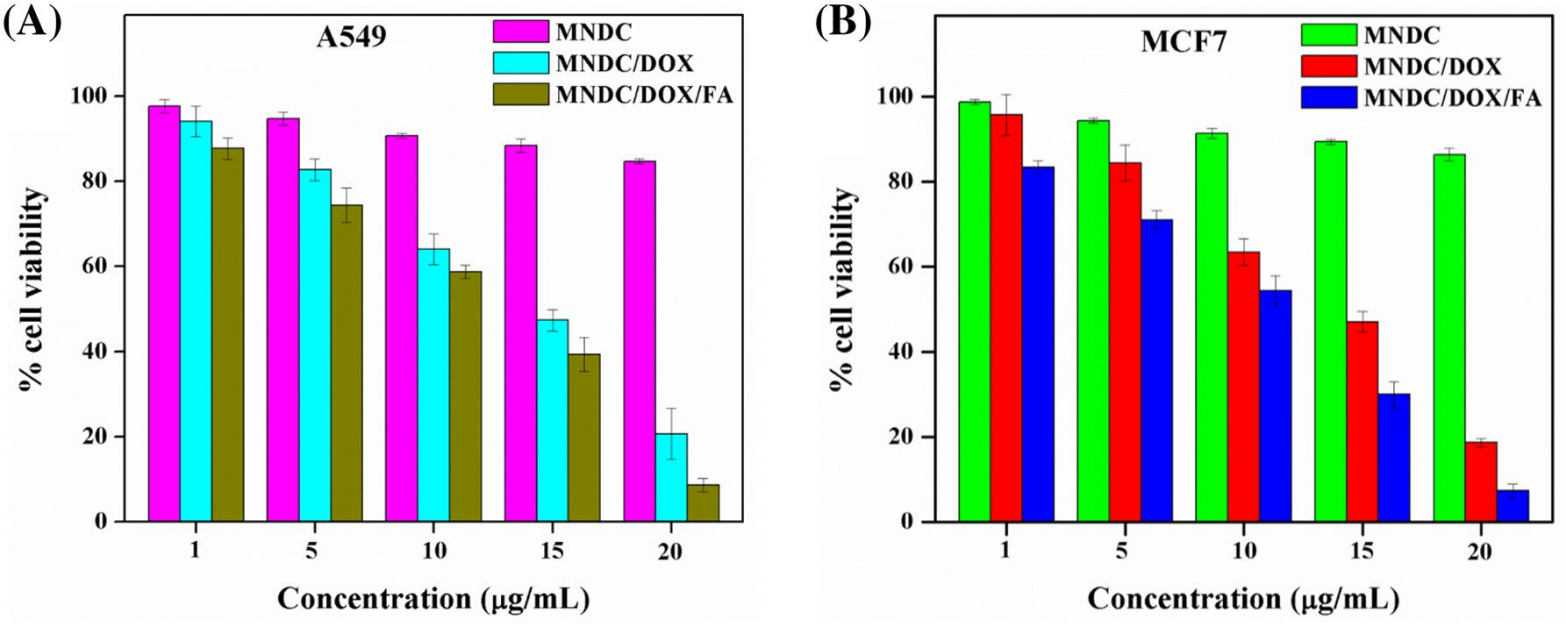
Relative cell viability of magnetic nano drug carrier (MNDC)/doxorubicin (DOX) and MNDC/DOX/folic acid (FA) loaded magnetic nano drug carrier (MNDC) against (**A**)—A549 and (**B**)—MCF-7 cancer cells at different concentrations (P < 0.05).

To investigate the cytotoxicity and selective targeting of the rGO/Fe_3_O_4_/CS/DOX/FA nanocomposite, A549 and MCF7 (cancer) cell lines were incubated FA-free or FA-loaded rGO/Fe_3_O_4_/CS/DOX/FA nanocomposites in vitro*.* The cell viability was 20.6% and 8.6% (A549 cells; Fig. 9A), and 18.6% and 7.3% (MCF7 cells; Fig. 9B) for the rGO/Fe_3_O_4_/CS/DOX/FA and rGO/Fe_3_O_4_/CS/DOX nanocomposites, respectively, at 1 mg/mL concentration. Besides, cell viability was reduced with the DOX-loaded rGO/Fe_3_O_4_/CS/FA nanocomposite at a lower 200 mg/mL concentration. Based on these results, the anticancer activity of the released DOX from the nanocomposite was unaffected. Furthermore, A549 cells had lower viability than MCF7 cells after incubation with the vehicles. It shows that the growth inhibition due to rGO/Fe_3_O_4_/CS/FA was larger in the tumor cells than the normal cells, which can be attributed to the folate receptor-mediated specific endocytosis. As shown in Fig. 9A,B, rGO/Fe_3_O_4_/CS and rGO/Fe_3_O_4_/CS/FA nanocomposites showed no obvious cytotoxicity on any material, even after a 48 h incubation, which demonstrated that rGO/Fe_3_O_4_/CS had excellent cytocompatibility.

Cancer cells can develop multi-drug resistance (MDR) after treatment with free DOX due to the p-glycoprotein pump's expression in the cell membrane. Due to this expression, the drug molecules can be pumped from the cytosol to the extracellular regions. However, the MDR effect can be avoided when tumor cells uptake polymeric NPs-containing drugs. Even though free DOX is transported into cells by a passive diffusion mechanism, the DOX-loaded rGO/Fe_3_O_4_/CS/FA nanocomposite is transported by the receptor-mediated endocytosis mechanism. Thus, rGO/Fe_3_O_4_/CS/DOX/FA is more readily absorbed than free DOX^42^. The inhibition of cancer cell growth is increased synergistically by rGO/Fe_3_O_4_/CS/DOX/FA. Targeted drug delivery with magnetic nanocarriers facilitates the active transport of drugs into specific regions, resulting in increased drug concentrations at the tumor region compared to routine systemic chemotherapy^43^.

#### Cellular uptake, multimodal imaging, and toxicity studies of the rGO/Fe_3_O_4_/CS/DOX/FA nanocomposite

**Figure 10:**
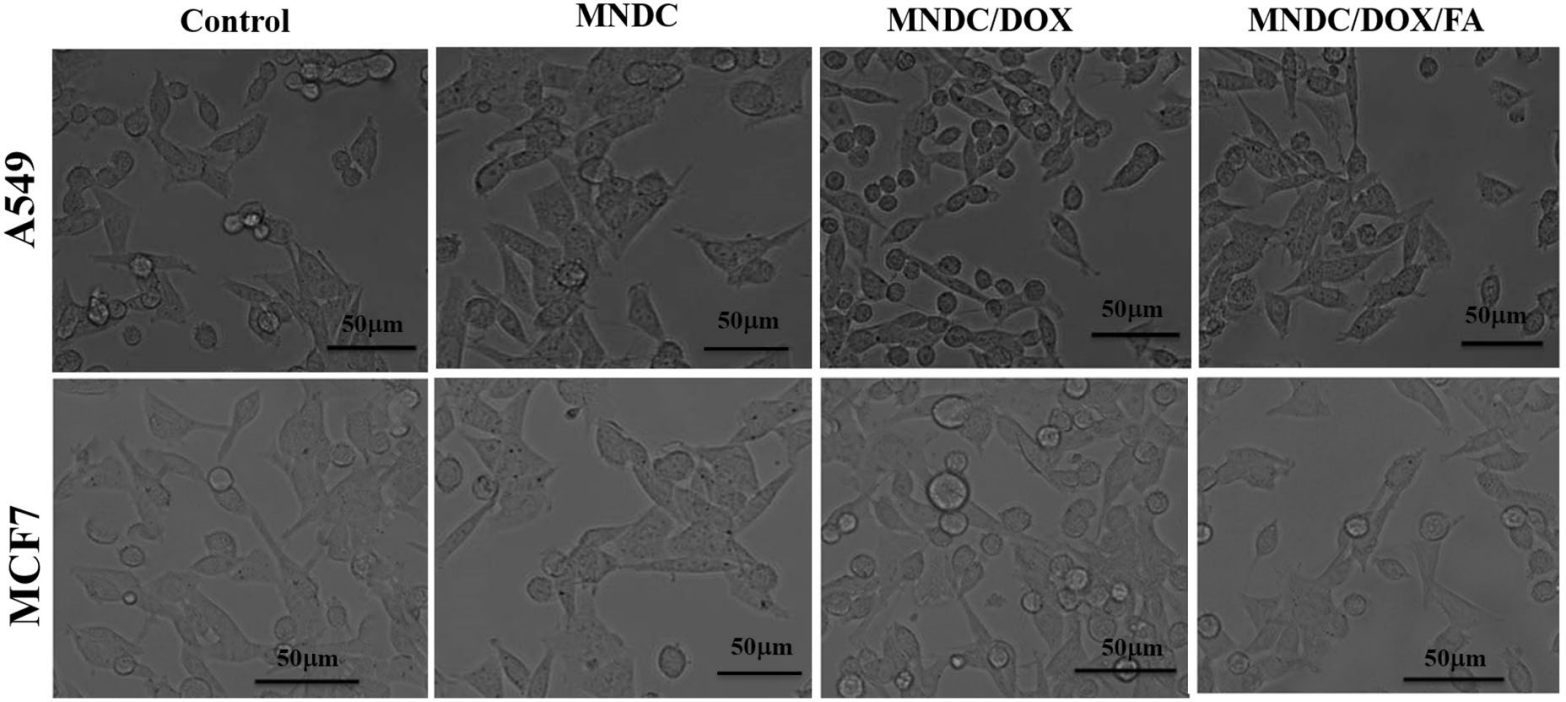
Cellular uptake and localization of rGO/Fe_3_O_4_/CS/DOX (magnetic nano drug carrier (MNDC)/doxorubicin (DOX)) and rGO/Fe_3_O_4_/CS/DOX/FA (MNDC/DOX/folic acid (FA)) in A549 and MCF-7 cancer cells observed by bright field emission images.

**Figure 11:**
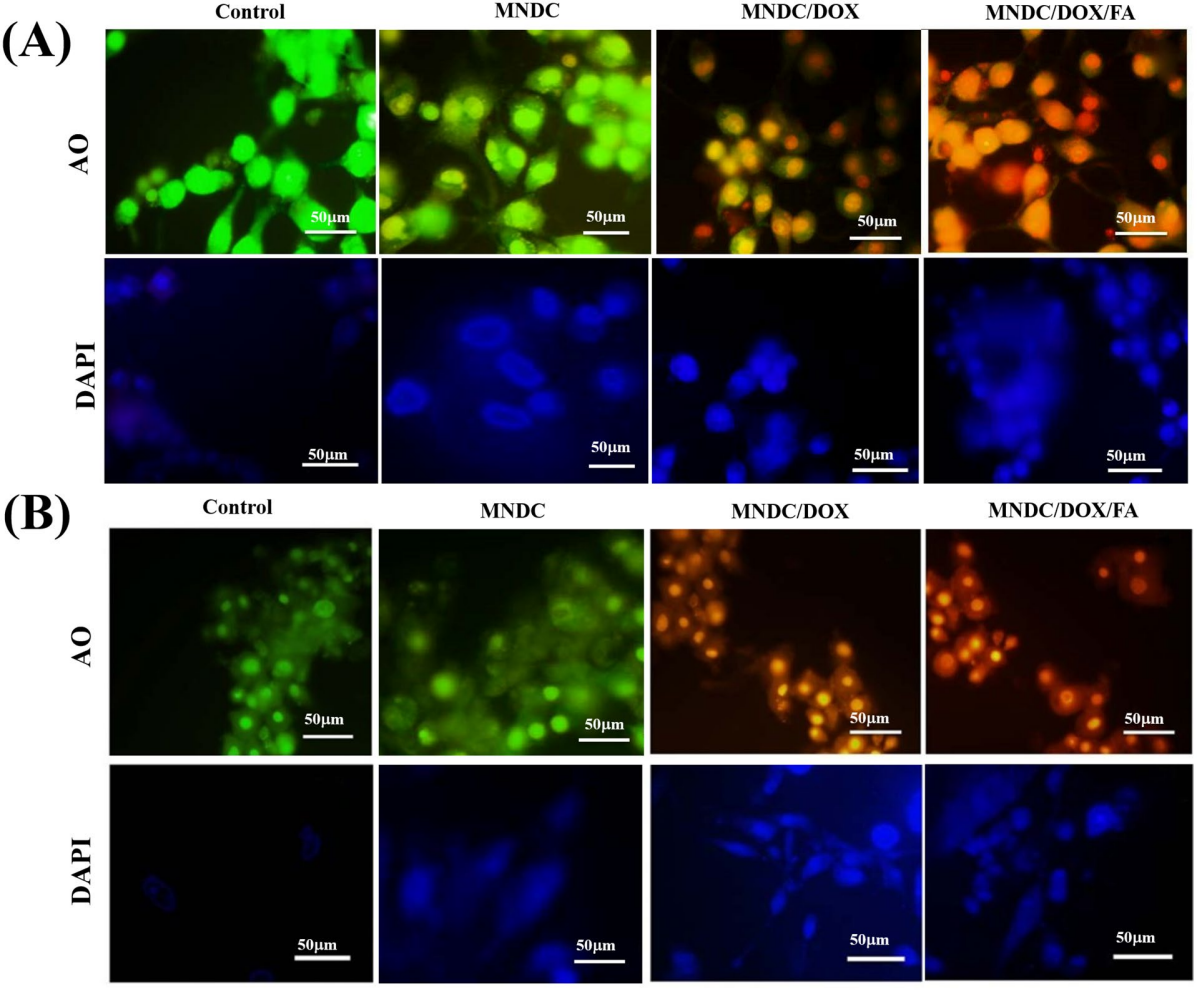
Cellular uptake and localization of rGO/Fe_3_O_4_/CS/DOX (magnetic nano drug carrier (MNDC)/doxorubicin (DOX)) and rGO/Fe_3_O_4_/CS/DOX/FA (MNDC/DOX/FA) in (**A**)—A549 and (**B**)—MCF-7 cancer cells observed by fluorescence microscopy images.

The DOX release from the nanocomposite and cell nucleus entry is clearly shown in Fig. 10. The bright field emission images of cells incubated with the rGO/Fe_3_O_4_/CS (magnetic nano-drug carrier (MNDC), rGO/Fe_3_O_4_/CS/DOX (magnetic nano-drug carrier (MNDC)/DOX) and rGO/Fe_3_O_4_/CS/DOX/FA (MNDC/DOX/FA) nanocomposites are shown (Fig. 10). DOX is a fluorophore, and its fluorescence emission can evaluate the intracellular activity of rGO/Fe_3_O_4_/CS, rGO/Fe_3_O_4_/CS/DOX and DOX-loaded rGO/Fe_3_O_4_/CS/FA nanocomposite with the A549 and MCF7 cell lines (Fig. 11A,B). The yellow fluorescence from the DOX emissions suggested the significant uptake of rGO/Fe_3_O_4_/CS/DOX/FA (DOX-loaded folate labeled particles) by the cells. The blue fluorescence, which represents DAPI, clearly indicated that rGO/Fe_3_O_4_/CS/DOX/FA was primarily localized in the nuclear region. Acridine orange (AO) was utilized to investigate DOX's localization in the perinuclear region and the nucleus. Under blue excitation, the green emission from DOX indicated its binding to the nucleus and cytoplasm^44^.

However, the drug-loaded nanocomposite was primarily internalized in the perinuclear region or in the nucleus, as shown in the image (Fig. 11). The AO fluorescent stain was used to observe the morphological changes in the apoptotic and necrotic cells. The green fluorescence and the yellow emission indicated that the nanocomposites entered into cells with broken membranes, such as apoptotic and dead cells, certain to targeted DNA fragments or apoptotic bodies. Quantitively, 80% of the cells were apoptotic and necrotic due to the DNA fragment injuries.

Furthermore, to corroborate the folate receptor-mediated drug delivery behavior, we compared the emission of rGO/Fe_3_O_4_/CS and rGO/Fe_3_O_4_/CS/DOX (folate unlabeled nanocomposite) to that of rGO/Fe_3_O_4_/CS/DOX/FA. The loading efficiency for the rGO/Fe_3_O_4_/CS/DOX/FA nanocomposite was 98%. The rGO/Fe_3_O_4_/CS/DOX/FA nanocomposite had higher uptake into the cancer cells, as these particles were conjugated with folic acid and cancer cells over-express folate receptors. Thus, the application of folic acid-conjugated Fe_3_O_4_ NPs for drug carriers could remarkably improve the uptake of DOX in cancer cells that over-express folate receptors. Based on these results, rGO/Fe_3_O_4_/CS/DOX/FA improved therapeutic efficacy by the synergistic effect of specific targeting, effective drug delivery, and improved anticancer activity of DOX. This mechanism is based on classical chemotherapy and the modern nanotechnology with rGO/Fe_3_O_4_/CS superparamagnetic NPs. Additionally, these nanocomposites can also treat the cancer cells by hyperthermia and photodynamic therapy and facilitate tumor imaging via their fluorescence property.

### Antibiofilm activity

**Figure 12:**
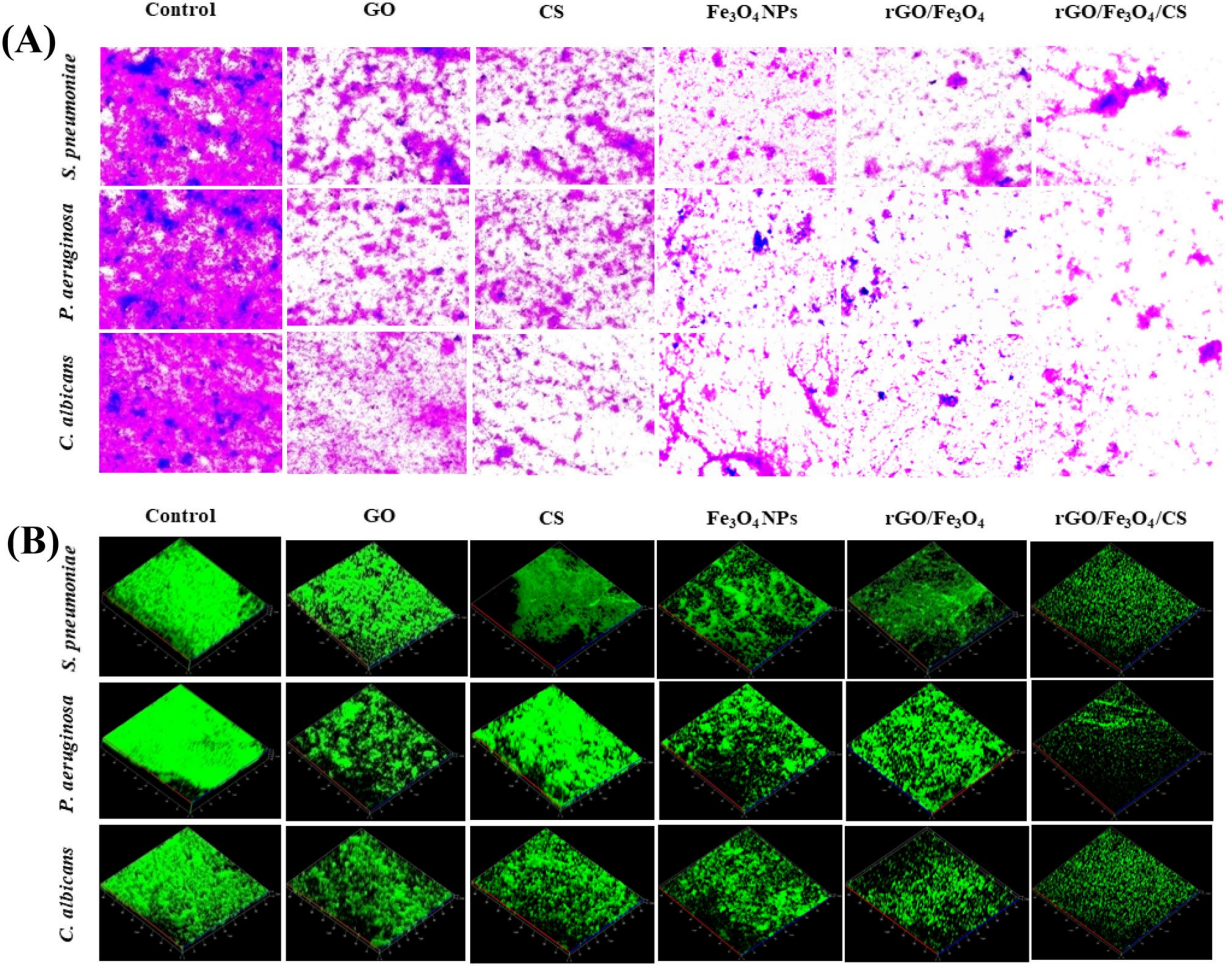
(**A**)—Light microscopy images of biofilms with crystal violet strain, (**B**) confocal laser scanning microscopy (CLSM) images of maturely formed microbial biofilms exposed to graphene oxide (GO), chitosan (CS), Fe_3_O_4_, rGO/Fe_3_O_4_ (5%), and rGO/Fe_3_O_4_/CS (15%) at 37 °C for 24 h. In the CLSM images, live microbes emit green light, while the dead cells were not colored.

The binding structure of AO to adherent cells caused by the in vitro biofilm formation of GO, CS, Fe_3_O_4_ NPs, rGO/Fe_3_O_4_, and rGO/Fe_3_O_4_/CS nanocomposites was observed using confocal laser scanning microscopy (CLSM). The bacteria *Streptococcus pneumoniae* (*S. pneumoniae), Pseudomonas aeruginosa* (*P. aeruginosa),* and fungus *Candida albicans (C. albicans),* have been studied in detail concerning their ability to form biofilms^8^. The Fe_3_O_4_ NPs with a minimum inhibitory concentration (MIC) of 50 μg had a minimal effect compared to the control on the biofilm structure and the proportion of the live bacteria in the biofilm after a 12 h incubation. After a 24 h incubation period with Fe_3_O_4_ NPs, a decrease in biofilm formation was observed to increase NPs concentration (Fig. 12; 74% inhibition of biofilm was observed for the rGO/Fe_3_O_4_/CS nanocomposite, while 63% inhibition of biofilm was observed for the rGO/Fe_3_O_4_ nanocomposite.

However, there were still quite a lot of biofilm structures remaining and the number of dead microbes significantly increased, indicating the biofilm ability of NPs. Results show that NPs induce the biofilm formation by *P. aeruginosa, S. aureus,* and *C. albicans;* however, the concentration of the NPs should be continuously increased for the biofilm to be destroyed. Therefore, the rGO/Fe_3_O_4_/CS nanocomposite destroyed a maturely formed biofilm structure and controlled the thickness by breaking the exopolysaccharides.

### Antioxidant activity

**Figure 13:**
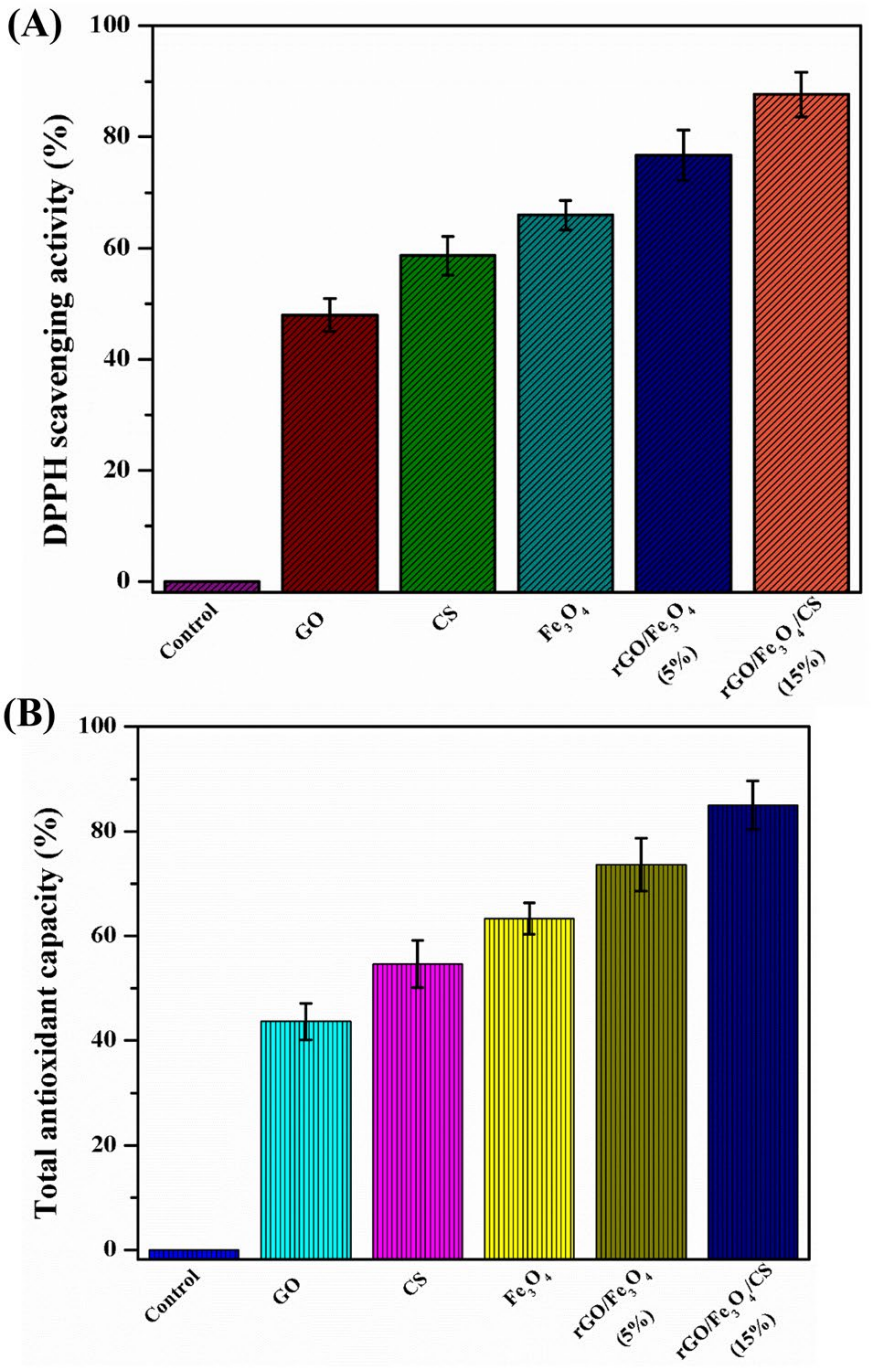
(**A**)—Total antioxidant capacity (μg/mg) and (**B**)—% radical 2,2-diphenyl-1-picrylhydrazyl (DPPH) scavenging activity of graphene oxide (GO), chitosan (CS), Fe_3_O_4_, rGO/Fe_3_O_4_ (5%), and rGO/Fe_3_O_4_/CS (15%) (P < 0.05).

The 2,2-diphenyl-1-picrylhydrazyl (DPPH) assay was utilized to investigate the nanomaterials' antioxidant activity in which ascorbic acid was considered positive control. DPPH is a stable compound that accepts hydrogens or electrons, and the reduction of DPPH is directly proportional to the samples' antioxidant nature. The free-radical scavenging ability of GO, CS, Fe_3_O_4_ NPs, rGO/Fe_3_O_4_ nanocomposite, and rGO/Fe_3_O_4_/CS nanocomposite as compared to the material combination as shown in (Fig. 13A,B). It reveals that both materials show free radical scavenging ability dependent on their materials. The free radical % scavenging potential of control, GO, CS, Fe_3_O_4_ NPs, rGO/Fe_3_O_4_ nanocomposite, and rGO/Fe_3_O_4_/CS nanocomposite were 0%, 48%, 58.6%, 66%, 76.6%, and 87.6%, respectively, based on the DPPH activity (Fig. 13A,B). The nanomaterials showed enhanced scavenging activity, increasing DPPH scavenging potential for CS, Fe_3_O_4_ NPs, and rGO. Lower concentrations of the polymer-coated nanocomposite reflected its higher potency for free radical scavenging and total antioxidant capacity. Similar trends in the improvement of DPPH scavenging behavior by GO, Fe_3_O_4_ NPs, and CS NPs have been previously reported^45–48^.

## Conclusions

We have developed a hybrid system by the solvothermal method that was highly biocompatible, water dispersible, fluorescent, superparamagnetic, and multifunctional. This hybrid possesses a large loading capacity of approximately 0.448 mg/mL^−1^ with the DOX anticancer drug. The release of DOX can be pH-triggered and controlled magnetically. Specifically, the surface modification of Fe_3_O_4_ NPs with folic acid improved its uptake by cancer cells. The toxicological study revealed the cytocompatible nature of rGO/Fe_3_O_4_/CS nanocomposite in A549 and MCF7 cells. In vitro cellular imaging of rGO/Fe_3_O_4_/CS/DOX/FA in A549 and MCF7 cells showed significant co-localization to the cytoplasmic region. The toxicity studies showed high biocompatibility of the rGO/Fe_3_O_4_/CS to zebrafish without inducing significant abnormalities.

Additionally, the zebrafish survival rate was not affected. Uniform distribution over the entire body was observed zebrafish using In vivo whole-animal imaging of rGO/Fe_3_O_4_/CS. Besides, rGO/Fe_3_O_4_/CS nanocomposite highly efficient in disrupting the biofilm formed by *S. aureus, P. aureginosa* and *C. albicans* and also exert productive antioxidant potential. Hence, rGO/Fe_3_O_4_/CS may serve as a biologically potent multipurpose material for future biomedical investigations and could be applied as a nano-deliver agent (nanocarrier) to the combination of photodynamic therapy with gene delivery or magnetically-guided drug.

## Materials and methods

### Chemicals and reagents

Graphite powder, ferric chloride (FeCl_3_.6H_2_O) (99%), ferrous sulfate (FeSO_4_.7H_2_O) (99.5%), sodium nitrate (NaNO_3_) (99%), sodium hydroxide (NaOH) (97%), potassium permanganate (KMnO_4_) (99%), sulfuric acid (H_2_SO_4_) (95–97%), folic acid (FA) (97%), glutaraldehyde (98%), sucrose (99%), and ethylene glycol (EG) (99%) were purchased from Merck (India). Chitosan (CS) powder (medium molecular weight- 190,000 to 310,000 Da) with a deacetylation degree of 85%, and doxorubicin hydrochloride (98% with 2 years of validation days) were purchased from Sigma-Aldrich (India). Microbial cultures (*Streptococcus pneumoniae* (MTCC 1936), *Pseudomonas aeruginosa* (MTCC 2642) and *Candida albicans* (MTCC 3959) were received from Microbial Type Culture Collection and Gene Bank (MTCC), Chandigarh, India and all media were supplied by Himedia (India). The analytical grade of chemical was used. The entire work was carried out using Milli-Q water.

### Material synthesis

#### Preparation of graphene oxide

Modified Hummer’s method was used to prepare to GO. The graphene oxide was prepared based on the Hummer’s method with some modification. Briefly, graphite powder (2 g) and NaNO_3_ (2 g) were mixed to 100 mL of concentrated sulfuric acid. After stirring the mixture in an ice bath for half an hour, 7 g of KMnO_4_ was added to the solution. Afterward, the solution was further stirred for 1 h at ambient condition. Next, the solution was stirred overnight in order to oxidize the graphite. One hundred milliliters of Milli-Q water was poured into the mixture to neutralize. An additional 400 mL of Milli-Q water was added, and the mixture was stirred for 30 min. Next, 10 mL of 30% hydrogen peroxide was added to terminate the reaction. Finally, the solution was subjected to stirring, centrifugation, and then washing (with 5% HCl) until the pH became close to 7. The resultant was separated by filtration and extensively washed with H_2_O; the obtained GO was then freeze-dried.

#### Synthesis of Fe_3_O_4_ NPs

The solvothermal method was utilized to prepare Fe_3_O_4_ NPs. FeCl_3_.6H_2_O (2 mM), FeSO_4_.7H_2_O (1 mM), and sucrose (0.1 mM) were added to 50 mL of ethylene glycol and dissolved by stirring vigorously. Subsequently, pH was adjusted by adding NaOH dropwise into the solution and further stirred for 30 min. As soon as the color changed from orange to black, a black precipice was noticed. The resuting solution was immediately transferred to a Teflon-lined stainless autoclave at 150 °C for 24 h. The mixture was allowed to reach room temperature by slow cooling. The resulting precipitate containing the Fe_3_O_4_ NPs was harvested using a permanent magnet discarding the supernatant. The precipitate was washed thrice with Milli-Q water and acetone. Finally, the precipitate was freeze-dried.

#### Synthesis of the rGO/Fe_3_O_4_ nanocomposite

A dispersion of GO (10 mg) in Milli-Q water (10 mL) and ethylene glycol (10 mL) was prepared by ultrasonication for 3 h. Then, 0.1 M ferric chloride, 0.05 M ferrous sulfate and sucrose were added to the dispersion while stirring the dispersion vigorously. Then, the pH of the mixture (12) was adjusted by the drop addition of NaOH. Following this, ascorbic acid (10 mg) was added as a reducing agent under continuous stirring for 1 h. The mixture was transferred into a stainless steel autoclave at 150 °C for 24 h. The solution was centrifuged to separate and wash the resulting nanocomposite (thrice)with water/acetone and freeze-dried.

#### Preparation of amphiphilic polymer rGO/Fe_3_O_4_/CS nanocomposite

The biocompatible rGO/Fe_3_O_4_ nanocomposite was fabricated by grafting the chitosan's functional groups on the NP surface. Under vigorous stirring, the CS powder was dissolved in 2% acetic acid at 25 ± 2 °C for about 2 days, and the solution was neutralized. To introduce CS functionalization, 10 mL of rGO/Fe_3_O_4_ nanocomposite (0.3 mg /mL) was mixed with 5 mL of CS (40 mg/mL with different concentrations 5, 10, and 15%) and sonicated for 30 min. To this mixture, 3 mL of 1% glutaraldehyde was added and stirred at 1000 rpm for 24 h. Hydrophilic and biocompatible rGO/Fe_3_O_4_/CS nanocomposite was obtained from the solution, washed with Milli-Q water.

#### Material characterization techniques

The infrared spectral studies were carried out using a Perkin-Elmer FT-IR spectrophotometer using KBr pellet. The crystalline structural analysis was performed using a PW3040/60 X’pert PRO X-ray diffractometer with Cu Kα radiation and a wavelength of 1.54060 Å. Microstructures of the pure and nanocomposite were examined by FE-SEM (Model: Hitachi S-4500). The chemical compositional analysis was studied using the EDX. HR-TEM (Tecnai) was used to study the particle size and nature. The chemical state analysis was performed with XPS (Carl Zeiss ) using Al Kα excitation at 250 W. The room temperature magnetization was measured by a vibrating sample magnetometer (VSM, Lake Shore, Model-7410, USA). The samples were also analyzed by UV–Vis spectroscopy (Shimadzu UV/Vis 1800 spectrophotometer), and the absorbance was measured at 620 nm using a Thermo Multiskan ELISA multiwell plate reader (EX, USA). The morphology of the A549 and MCF7 cells was studied using Nikon (Japan) bright-field inverted light microscopy at 40X magnification and fluorescence microscope (Nikon Eclipse, Inc, Japan) at 400X magnification with an excitation filter at 480 nm. Biofilm inhibition was inspected by light microscopy (Olympus cx21i) CLSM (NIKON ECLIPSETS 100) at a magnification of 20X.

### Pharmacological Study

#### Microinjection of GO, CS, Fe_3_O_4_ NPs, rGO/Fe_3_O_4_ (5%), and rGO/Fe_3_O_4_/CS (15%) into zebrafish embryos and microscopic measurements

Wild-type AB strains of *Danio rerio* (*D. rerio-* zebrafish) embryos were supplied from the zebrafish core facility center, National Tsing Hua University. The MNDC samples were ultrasonicated for 30 min in Milli-Q water and then microinjected (10 nL) in the fresh embryos placed on the microinjection embryo tray. The dark incubated (28 °C) control embryos (without MNDC) and MNDC-loaded embryos were periodically observed using microscopic images^49,50^. The abnormality of infant fish was detected using a MicroFire camera (Olympus CX-21i Trinocular Microscope, LED illumination) mounted onto a Leica MZ16 stereomicroscope (Meyer Instruments, Houston, TX, USA).

### Targeted drug delivery

#### Preparation of folate conjugated rGO/Fe_3_O_4_/CS (15%) nanocomposite

For the sufficient attachment of folic acid to the rGO/Fe_3_O_4_/CS (15%) nanocomposite, we adapted a method reported by Guo et al*.* in which 20 mg of the rGO/Fe_3_O_4_/CS (15%) nanocomposite was washed and resuspended with dimethyl sulfoxide (DMSO). About 0.1 mg of FA and 0.05 μL of (3-aminopropyl) triethoxysilane (APTS) were dissolved in 1 mL of DMSO in a flask. Then, 0.03 mg of N-hydroxysuccinimide and 0.05 mg of 1-(3-dimethylaminopropyl)-3-ethylcarbodiimide hydrochloride were added to the flask and stirred for 2 h. In another flask, 4 mL of toluene was taken. The nanocomposite DMSO suspension and the folate- APTS solution were added to toluene. The solution was stirred for 20 h at ambient conditions. The folate conjugated nanocomposite was thoroughly centrifuged and rinsed with toluene, and vacuum-dried.

#### Preparation of drug encapsulated nanocomposite (rGO/Fe_3_O_4_/CS/FA/DOX)

The anticancer drug DOX was encapsulated in rGO/Fe_3_O_4_/CS and rGO/Fe_3_O_4_/CS/FA in various concentrations by a simple mixing technique to initiate physisorption^51^. Initially, 10 mg of DOX were mixed in 5 mL of PBS buffer with pH 7.4 and then dispersed by ultrasonication for 10 min. Various concentrations of DOX (0.05, 1, 1.5, 2, 2.5, 3, 3.5, 4, 4.5 and 5% mg/mL) were prepared from the stock (10 mg in 5 mL) solution by serial dilutions (in triplicate), and were subjected to 2 min of sonication. To each of the drug suspensions, a known and fixed amount of rGO/Fe_3_O_4_/CS/FA (100 μg/mL) was added and incubated for 24 h. The drug-loaded nanocomposite was centrifuged to separate the free drug molecules at 12,000 rpm for 10 min and maintained at 4 °C before further analysis. The drug concentration was measured using UV–Vis spectra of the supernatant and comparison with a calibration curve. The amount of drug-loaded in the rGO/Fe_3_O_4_/CS/DOX/FA was determined by comparing the supernatant's concentration to that of the free drug molecules taken initially.

#### Kinetics of pH-dependent drug release

The pH-dependent DOX release from the rGO/Fe_3_O_4_/CS/ FA/DOX was analyzed by making a suspension of rGO/Fe_3_O_4_/CS/ FA/DOX with a constant content of DOX dissolved in PBS buffer at physiological (pH 7.4) and acidic (pH 5.5) pH levels. The quantity of drug released from the nanocomposite was estimated from the UV–vis absorption spectra of 1 mL of released PBS taken from both samples at regular time intervals. The samples were simultaneously maintained under an exact temperature of 37 °C in a shaking incubator. The volume of PBS withdrawn for analysis was replaced with the same volume of fresh PBS. All of the measurements were done in triplicates. The control graph measured the actual releasing rate at various pHs, and the drug content loaded into the rGO/Fe_3_O_4_/CS/ FA/DOX was calculated by equation.

#### Maintenance of cell lines

Experimental cell lines include A549 and MCF7 were supplied from the National Centre for Cell Sciences (NCCS), Pune, India. The cell lines were sustained in Dulbecco’s modified eagles medium (DMEM), holding 10% Fetal bovine serum (FBS). The cells were sustained in a humidified CO_2_ incubator according to procedure^42^.

#### Cytotoxic analysis

The toxic activity of MNDC, MNDC/DOX and MNDC/DOX/FA against cancer cell lines were studied using MTT [3-(4,5-dimethylthiazol-2-yl)-2,5-diphenyltetrazolium bromide] test^49^. Certain cancer cell lines (1 × 10^4^ cells/well) were introduced into the well of 96-well plate at their IC_50_ values. The medium was introduced with serially diluted MNDC, MNDC/DOX and MNDC/DOX/FA, and allowed to incubate for 48 h. After incubation, the medium was substituted with 100 µL of the MTT solution and incubated at 37 °C for 4 h. The plate was read at 620 nm in an ELISA multi-well plate reader (ThermoMultiskan EX, USA). The cell viability was calculated using the below formula, Cell viability (%) = Absorbance value of treated sample/ Absorbance value of control × 100.

#### Morphological analysis

Change in the morphology of the A549 and MCF-7 cancer cell lines (1 × 10^5^ cells/coverslip) concerning the release of drugs, which was probed using MNDC, MNDC/DOX and MNDC/DOX/FA. The coverslips presented on the clean glass slides were detected using a Nikon (Japan) bright-field inverted light microscopy at 40X magnification.

### Apoptotic analysis

The cell suspension (1 × 10^5^ cells/mL) was stained using ethidium bromide (EthBr) (100 mg/mL) and AO and mildly spread on the slide to investigate apoptosis. The cancer cell lines were gently flooded with Na_2_HPO_4_–KH_2_PO_4_ buffer solution (pH 7.2) before AO/EtBr staining. Afterward, the cells were rinsed with PBS twice and evaluate under a fluorescence microscope at 40X magnification with an excitation filter at 480 nm. The (4′,-6-diamidino-2-phenylindole) DAPI analysis was conducted based on the same protocol^52^.

### Antibiofilm assessment

The samples' potential antibiofilm ability was investigated using *S. pneumonia, P. aeruginosa,* and *C. albicans* as Gram-positive, Gram-negative, and fungus, respectively. Briefly, The fungal and bacterial biofilm was grown on the sterile glass submerged potato dextrose broth and nutrient broth, respectively. Afterward, the nanomaterials were individually added to each well and incubated at 37 °C for 24 h. Consequently, the loosely attached cells on the glass were thoroughly removed using PBS, and the glass was stained nucleic acid fluorescent dye AO (0.4%). Finally, samples were evaluated using under CLSM (Carl Zeiss LSM 710) with a 488 nm argon laser using a BP 500–640 bandpass emission filter Zen 2009 software (Carl Zeiss, Germany)^53^.

### Antioxidant assay

Free radicals quenching property of GO, CS, Fe_3_O_4_ NPs, rGO/Fe_3_O_4_ (5%), and rGO/Fe_3_O_4_/CS (15%) were assessed using DPPH and reducing power method, as described below.

### DPPH radical scavenging analysis

DPPH was used to measure the antioxidant activity of GO, CS, Fe_3_O_4_ NPs, rGO/Fe_3_O_4_ (5%), and rGO/Fe_3_O_4_/CS (15%) based on a previously reported method^42^. DPPH stock was prepared in 40 mL of acetate buffer (0. 1 M, pH 5.5) with methanol(40 mL: 60 mL) and maintained for 30 min at 30 °C in dark conditions and detected at 520 nm. Ascorbic acid was considered as a positive control^53^.

### Reducing power analysis

The total reducing power of GO, CS, Fe_3_O_4_ NPs, rGO/Fe_3_O_4_ (5%), and rGO/Fe_3_O_4_/CS (15%) was assessed by the following method. Briefly, 0.1 mg/mL in 500 μL of d.H_2_O was mixed in 2.5 mL of phosphate buffer (0.2 mL, pH 6.6). To this solution, 2.5 mL of potassium ferricyanide (1%) was introduced and incubated for 20 min at 50 °C. Subsequently, 2.5 mL of 10% trichloroacetic acid (TCA) was mixed and incubated for 10 min. Afterward, 2.5 mL of d.H_2_O and 0.5 mL of ferric chloride (0.01%) were added with the supernatant and was detected at 700 nm in the UV–vis spectrometer against phosphate buffer as a blank^53^.

### Statistical assessment

The analysis was made with a one-way analysis of variance (ANOVA) with significance levels at p < 0.05. All data were shown as mean ± standard deviation values.
